# The causal association between resting state intrinsic functional networks and neurodegeneration

**DOI:** 10.1093/braincomms/fcaf098

**Published:** 2025-03-04

**Authors:** Malik Nassan, Iyas Daghlas, Bram R Diamond, Adam Martersteck, Emily Rogalski

**Affiliations:** Mesulam Center for Cognitive Neurology and Alzheimer’s Disease, Northwestern University, Chicago, IL 60611, USA; Department of Neurology, University of California San Francisco, San Francisco, CA 94143, USA; Mesulam Center for Cognitive Neurology and Alzheimer’s Disease, Northwestern University, Chicago, IL 60611, USA; Healthy Aging and Alzheimer’s Research Care (HAARC) Center, University of Chicago, Chicago, IL 60637, USA; Healthy Aging and Alzheimer’s Research Care (HAARC) Center, University of Chicago, Chicago, IL 60637, USA

**Keywords:** Genomics, causality, default mode network, cascading network failure, selective vulnerability

## Abstract

Alterations of resting state intrinsic functional networks have been associated with neurodegenerative diseases even before the onset of cognitive symptoms. Emerging hypotheses propose a role of resting state intrinsic functional networks alterations in the risk or vulnerability to neurodegeneration. It is unknown whether intrinsic functional network alterations can be causal for neurodegenerative diseases. We sought to answer this question using two-sample Mendelian randomization. Using the largest genome-wide association study of resting state intrinsic functional connectivity (*n* = 47 276), we generated genetic instruments (at the significance level 2.8 ×10^−11^) to proxy resting state intrinsic functional network features. Based on the known brain regions implicated in different neurodegenerative diseases, we generated genetically proxied resting state intrinsic functional features and tested their association with their paired neurodegenerative outcomes: features in parieto-temporal regions and Alzheimer dementia (111 326 cases, 677 663 controls); frontal region and frontotemporal dementia (2154 cases, 4308 controls); temporal pole region and semantic dementia (308 cases, 616 controls), and occipital region with Lewy body dementia (LBD) (2591 cases, 4027 controls). Major depressive disorder outcome (170 756 cases, 329 443 controls) was included as a positive control and tested for its association with genetically proxied default mode network (DMN) exposure. Inverse-variance weighted analysis was used to estimate the association between the exposures (standard deviation units) and outcomes. Power and sensitivity analyses were completed to assess the robustness of the results. None of the genetically proxied functional network features were significantly associated with neurodegenerative outcomes (adjusted *P* value >0.05), despite sufficient calculated power. Two resting state features in the visual cortex showed a nominal level of association with LBD (*P* = 0.01), a finding that was replicated using a different instrument (*P* = 0.03). The genetically proxied DMN connectivity was associated with the risk of depression (*P* = 0.024), supporting the validity of the genetic instruments. Sensitivity analyses were supportive of the main results. This is the first study to comprehensively assess the potential causal effect of resting state intrinsic functional network features on the risk of neurodegeneration. Overall, the results do not support a causal role for the tested associations. However, we report a nominal association between visual network connectivity and Lewy body dementia that requires further evaluation.

## Teaser

Nassan *et al*. leveraged Mendelian randomization to evaluate whether genetically predicted brain connectivity patterns can influence the risk for neurodegeneration later in life. Although the results overall did not support this hypothesis, they report a possible association between the visual network connectivity and Lewy body disease, which requires further study.

## Graphical Abstract

**Graphical Abstract:**
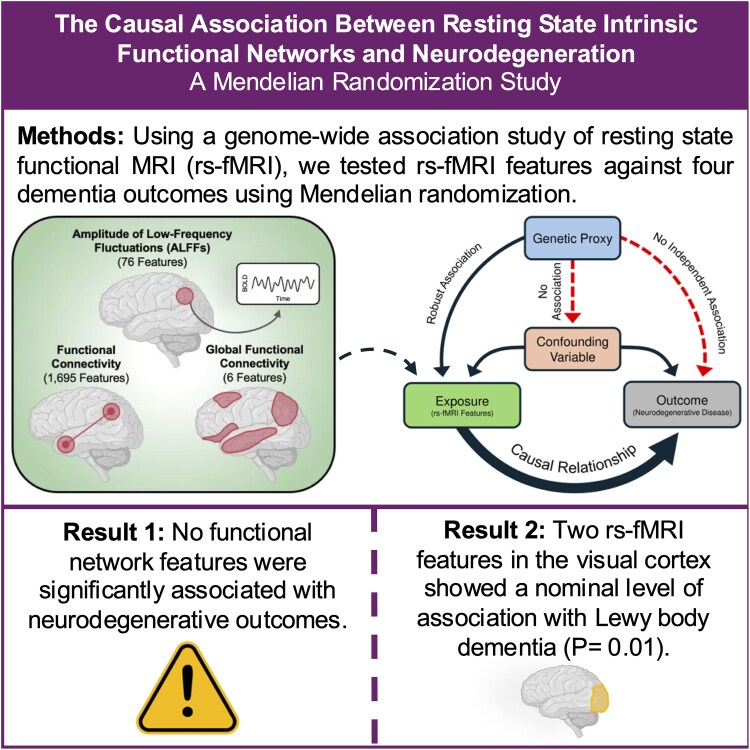

## Introduction

Neurodegenerative disorders are among the leading causes of disability worldwide.^1,2^ Understanding disease pathogenesis and its causal factors is critical to the development of effective treatments to prevent or reverse the process of neurodegeneration. Functional neuroimaging, including resting state functional MRI (rsfMRI), has been applied to further our conceptualization of the pathogenesis of neurodegenerative diseases.^3^ rsfMRI records fluctuations in local blood oxygen-level dependent signal, which are closely coupled to neural activation and can be used to provide a valuable estimate of neural network connectivity. Using rsfMRI, Alzheimer’s dementia (Ad, used broadly here to encompass clinical and pathological diagnosis) and other neurodegenerative disorders have been characterized by distinctive disease-related functional topographies.^3^ In addition, abnormal network connectivity correlates with clinical dementia syndrome, symptom severity, and can be modulated by clinical interventions.^3^

Alterations of resting state intrinsic functional network connectivity have been observed at early stages of neurodegenerative diseases, even before the onset of cognitive symptoms.^3^ Recently, cascading network failure has been proposed as a potential inciting factor for the neurodegeneration cascade, even before detectable amyloid levels in Ad.^4^ This model was supported by the finding that in Ad patients, the posterior default mode network (DMN) fails before measurable amyloid plaques; and seems to initiate connectivity cascade changes that continue throughout the disease spectrum.^4^ However, it is not known if such connectivity pattern changes are the inciting event driving downstream molecular events related to synaptic activity embedded in these systems. It has been argued that in Ad, the accumulation of beta-amyloid plaques would accelerate the preexisting functional degradation of the DMN, which has been associated with aging.^5-9^ On the other hand, selective vulnerability has been proposed for why specific neurodegenerative disorders or their variants affect specific neural networks and cognitive functions.^10-12^ For example, it has been proposed that early-life neurodevelopmental changes could create a locus of least resistance for neurodegenerative disease to target.^13,14^ Alterations in functional connectivity have been associated with changes in neuroplasticity^15^ seen early in the neurodegeneration process and correlate with variation in microglial-modulated synaptic degeneration and cognitive impairment.^16-19^ As such, alterations in functional connectivity may reflect underlying neuropathological changes that contribute to neurodegeneration.

Presently, it is not known whether alterations of resting state intrinsic functional networks, such as connectivity changes in the DMN, are independently causal for neurodegeneration. This question is of significant clinical relevance because it could add to our understanding of the inciting process of the neurodegeneration cascade. In addition, functional connectivity as a potential risk factor may be modifiable by non-invasive interventions such as transcranial magnetic stimulation.^20^ Thus, studying the potential causal association between resting-state functional connectivity and neurodegenerative diseases can significantly impact future research and clinical practice. Observational studies are limited in their ability to answer such questions because rsfMRI measurements need to be collected decades before the onset of the cognitive decline, neuropathological burden (such as amyloid or phospho-tau), and any level of neurodegeneration. Furthermore, observational studies can be confounded by shared risk factors (e.g. *APOE4* can influence both rsfMRI functional connectivity and Ad risk),^21^ and by bias due to reverse causality.

Mendelian randomization (MR) is a statistical genomic approach that is ideal for addressing this question since it leverages the random assortment of genetic variants at gametogenesis to proxy causal impacts of exposures on disease outcomes.^22^ This approach minimizes potential bias by residual confounding and reverse causality.^23^ The MR approach has made significant contributions in neurology and can provide answers to causal associations with neurological diseases.^24^ We leveraged the MR approach to test the causal effect of genetically proxied rsfMRI intrinsic networks (a heritable phenotype)^25,26^ on risk of Ad and other main neurodegenerative disorders [frontotemporal dementia (FTD), semantic dementia (SD), and Lewy body dementia (LBD)]. This study can be conceptualized as a natural experiment whereby random assignment at birth to lifelong intrinsic functional network patterns is tested for its causal effect on neurodegeneration risk later in life. In other words, this study is attempting to answer the following question: ‘Do alterations in functional connectivity (measured by rsfMRI) cause neurodegeneration?’

## Materials and methods

### Ethical approval and patient consent

This study used publicly available GWAS summary statistics, and therefore institutional review board approval was not required. The original cohorts in the included GWAS studies have consented their participants per their institutional guidelines.^27-30^

### Study design

This study utilized a two-sample MR approach using GWAS summary statistics data.^23^ We selected genetic instruments to proxy resting state intrinsic functional networks features as exposures (from the largest to date rsfMRI GWAS)^27^ and tested their causal relationship with four types of neurodegenerative disease outcomes: Ad, FTD, SD and LBD (Table 1).

**Table 1:** Summary of the exposures and outcomes sources and their top GWAS hits

	Study	GWAS phenotype	Cases	Controls	Number of significantly associated SNPs
Exposure	Bingxin Zhao *et al*.^27^ *Nature Genetics*	Intrinsic functional networks connectivity	47 276 individuals		45
Outcomes	Céline Bellenguez *et al*.^28^ *Nature Genetics*	Ad	111 326	677 663	75
	Raffaele Ferrari *et al*.^29^ *Lancet Neurology*	FTD whole cohort	2154	4308	3
		SD	308	616	0
	Ruth Chia *et al*.^30^ *Nature Genetics*	LBD	2591	4027	5

Ad, Alzheimer’s dementia; FTD, Frontotemporal dementia; LBD, Lewy body dementia; SD, Semantic Dementia; SNP, single nucleotide polymorphism.

**Figure 1:**
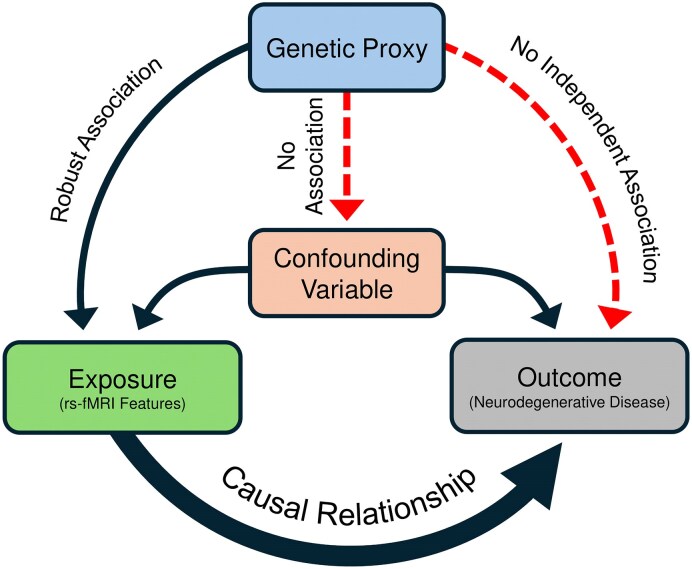
**Assumptions of the MR study design.** rs-fMRI, resting state functional MRI.

The MR study design is based on three assumptions: (i) the genetic instruments of intrinsic functional networks connectivity are strongly associated with the exposure of interest at a genome-wide level of significance, selected here as *P* < 2.8 × 10^−11^, (ii) the associations of the genetic instruments with the exposure are not confounded, and (iii) the genetic instruments affect the risk of the outcomes only through the exposure of interest, intrinsic functional networks connectivity (Fig. 1). Our study implemented quality control measures to assure the validity of the above assumptions and was conducted following the STROBE-MR guidelines for transparent reporting of MR analyses (STROBE-MR checklist).^31^

### Genetic associations with exposures

#### Selecting the neuroimaging features of interest

The primary inputs for this study were genetic instruments for the exposures selected from the largest rsfMRI GWAS (*n* = 42 276 participants) completed by Zhao *et al*.^27^ The Zhao *et al*.^27^ study tested associations of 1777 rsfMRI features (76 fluctuation amplitude nodes, 1695 functional connectivity features, 6 global connectivity) with 9 026 427 common genetic variants in a European sample.^27^ Zhao *et al*.^27^ defined fluctuation amplitude node features as the temporal standard deviation within a given component. Zhao *et al*.^27^ defined functional connectivity as the synchronized connectivity between two nodes using Independent Component Analysis (ICA) with dual regression. Zhao *et al*.^27^ defined global connectivity features as the top six components selected from the adjacency matrix of all nodes by principal-component analysis and ICA. Zhao *et al*.^27^ found 191 of the 1777 features were significantly associated with 45 genetic regions, *P* < 2.8 × 10^−11^, calculated based on the total number of the tested associations between the genetic variants and imaging phenotypes in that GWAS analysis.^27^

**Figure 2:**
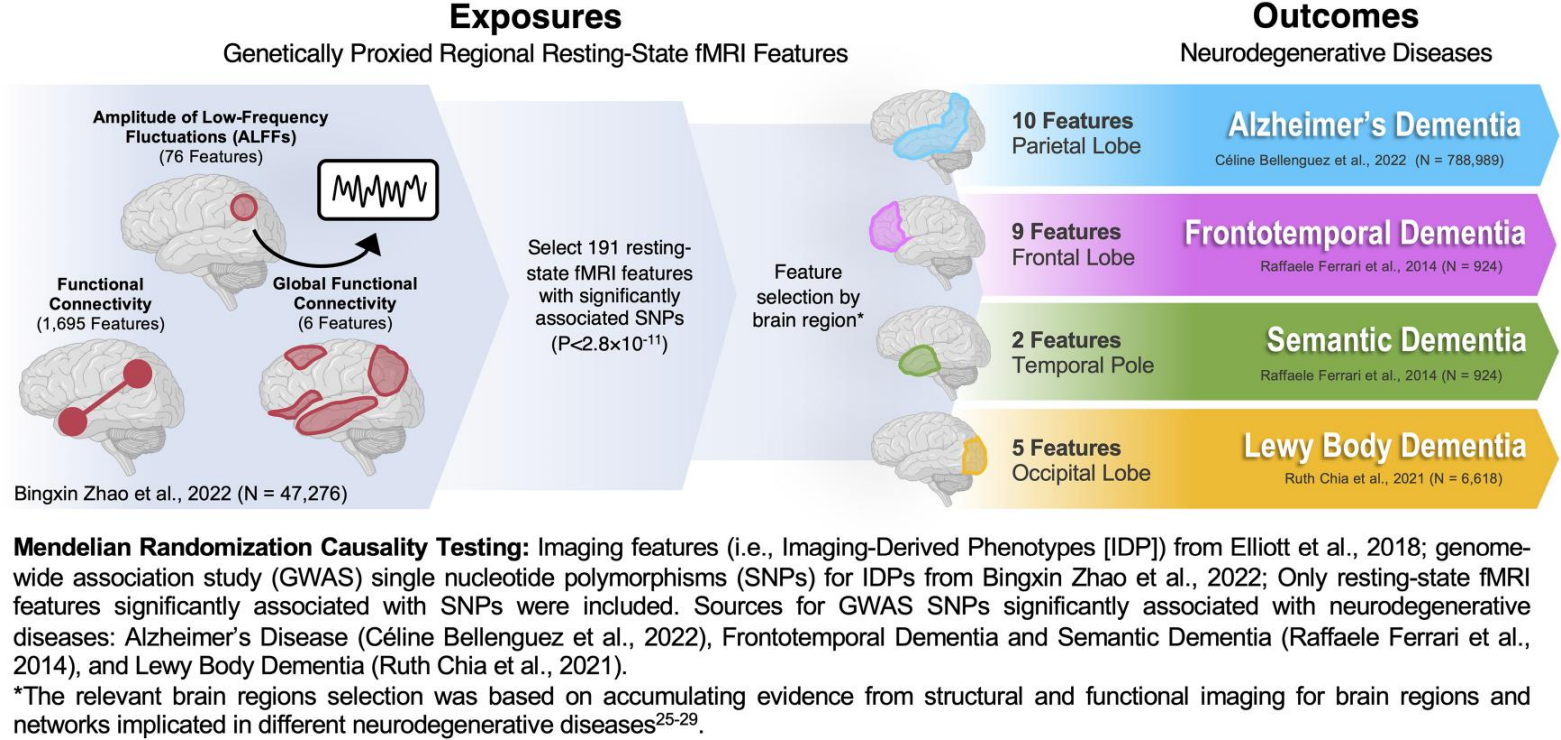
**Schematic illustration explaining the selection of exposures and outcomes for the MR analyses.** fMRI, functional MRI.

Our study queried a 26 of the 191 features based on a literature-informed^32-36^  *a priori* regions of interest (ROI) for each of the four neurodegenerative dementias included in this study. The anatomical locations for nodes were defined based on their proximity to the automated anatomical labelling atlas.^37^ A parieto-temporal ROI was used for the Ad group, and 10 features, comprised of 8 fluctuation amplitude nodes, 1 functional connectivity pair, and 1 global connectivity feature were queried. A frontal ROI was used for the FTD group and 9 features comprised of 9 fluctuation amplitude nodes. A temporal Pole ROI was used for SD, and two features, comprised of 2 nodes were queried. An occipital ROI was used for LBD, and five rsfMRI features, comprised of 5 nodes were queried (Fig. 2, Table 2). Information about the rsfMRI data acquisition and processing as well as rsfMRI GWAS analyses completed by Zhao *et al*.^27^ is summarized (Supplementary Notes).

**Table 2:** Selection of the exposures to be tested for association with the outcomes

Exposure category	Associated outcomes	Node/edge	Anatomical location	Network	Number of significantly associated SNPs (at LD 0.1)
DMN, parieto-temporal	Ad	Net25_Node20	Precuneus	Default_mode; Central_executive	15
		Net100_Node5	Precuneus; Angular; Middle_cingulate	Default_mode; Central_executive	7
		Net100_Node11	Angular; Middle_temporal	Default_mode; Central_executive	8
		Net100_Node24	Inferior_parietal; Angular	Central_executive; Attention	10
		Net100_Node36	Precuneus	Default_mode; Central_executive	10
		Net100_Node39	Precuneus; Superior_parietal	Attention; Central_executive	10
		Net100_Node49	Middle_temporal; Angular	Default_mode	5
		Net25_Node9	Inferior_parietal; Angular; Middle_temporal	Default_mode; Central_executive	17
		Net100_Pair5_45	(Precuneus; Angular; Middle_cingulate) ≤> (Superior_frontal; Middle_frontal)	(Default_mode; Central_executive) ≤> (Salience; Default_mode)	5
		Net_Edge_ICA2	NA	Central_executive; Salience; Default_mode	15
Frontal	FTD	Net25_Node16	Superior_frontal; Middle_frontal	Salience; Central_executive	15
		Net100_Node7	Superior_frontal; Anterior_cingulate	Default_mode; Limbic	7
		Net100_Node13	Superior_frontal	Default_mode	9
		Net100_Node15	Insula; Anterior_cingulate	Salience; Default_mode	14
		Net100_Node21	Superior_frontal	Default_mode; Central_executive	8
		Net100_Node31	middle_frontal	Central_executive	7
		Net100_Node37	Middle_frontal	Central_executive; Salience	11
		Net100_Node40	Superior_frontal; Middle_frontal	Central_executive	8
		Net100_Node45	Superior_frontal; Middle_frontal	Salience; Default_mode	8
Temporal pole	SD	Net100_Node9	Middle_temporal; Temporal_pole	Default_mode	7
		Net100_Node43	Inferior_temporal; Fusiform; Temporal_pole	Limbic; Default_mode	5
Occipital	LBD	Net100_Node4	Middle_occipital; Superior_occipital	Attention; Visual	7
		Net100_Node8	Calcarine; Lingual	Visual	5
		Net100_Node10	Middle_occipital; Precuneus; Calcarine	Default_mode; Central_executive	7
		Net100_Node38	Putamen; Caudate	Subcortical-cerebellum	7
		Net100_Node1	Calcarine; Lingual; Cuneus	Visual	4

Ad, Alzheimer’s dementia; DMN, default mode network; FTD, Frontotemporal dementia; LBD, Lewy body dementia; SD, Semantic Dementia; SNP, single nucleotide polymorphism.

#### Generating the genetic instruments of the exposures

Using the significant SNPs associated with neuroimaging features of interest from the discovery stage of the GWAS,^27^ we generated genetic proxies for 26 features (Table 2). MR relies on the assumption that there are no other causal pathways between the genetic variants used as a genetic instrument and the outcome of interest other than through the exposures being studied.^22,23^ One way to meet this assumption is to control for known pleiotropy by removing pleiotropic SNPs from the exposure instrument. Because APOE4 is a known risk factor for Ad,^38^ the APOE SNP significantly associated with rsfMRI (rs429358: part of the APOE4 haplotype) and the loci in Chromosome 19 in high LD (LD > 0.001) with this SNP were removed from the genetic instruments of the exposures to control for pleiotropy. The genetic instruments for all the features are listed in Supplementary Tables 1–4 only genetic variants with known rs SNP identification were included. Genetic instruments were then clumped for all exposures using clumping function in the R TwoSampleMR package (parameters: *R*^2^ 0.001, clumping window 10,000, and selecting the European subsample in the 1000 genomes reference panel). Ambiguous palindromic SNPs were removed from the genetic instruments.

Due to the nominal association later found between occipital nodes rsfMRI fluctuation amplitude and LBD, we assessed other instruments of visual network rsfMRI GWAS. Another recent GWAS performed genome-wide analyses of resting state network measures of intrinsic brain activity in about 36 150 European ancestry participants from the UK Biobank.^39^ The mean age at the time of brain scan was 63.6 years (SD of 7.5 years). Participants with a history of stroke or other major central nervous system (CNS) disease (e.g. multiple sclerosis, Parkinson disease, dementia or any other CNS neurodegenerative condition) were excluded (*n* = 707). This GWAS assessed the three visual networks (Visual 1, Visual 2 and visual association)^40^ mean within-network functional connectivity between each pair of components, which was calculated to give an overall measure of the connectivity for the network for each subject, and create a standard deviation effect size. Only Visual 1 (which involved the calcarine fissure and primary visual cortex) was significantly associated with genetic variants. Visual 1 network GWAS revealed three significantly associated loci in PLCE1, UFL1 and C10orf91. Those 3 SNPs were used as a secondary MR analysis for their association with LBD.

### Genetic associations with the outcomes

GWAS summary-level data for the largest GWAS studies of Ad, FTD, SD and LBD were used for the MR analyses (Table 1). The largest Ad GWAS study was included for analysis (111 326 cases, and 677 663 controls).^28^ That GWAS included consortia of participants with primarily European ancestry. Ad diagnoses were made based on either experts’ clinical diagnosis or through self-report. In addition, self-report of family history of Ad (only one sub-cohort) were included as cases. The study reported 75 genetic variants associated with Ad at a genome-wide level of significance. The summary statistics data for this GWAS was obtained from the GWAS Catalog portal (https://www.ebi.ac.uk/gwas/).

The largest GWAS for FTD was included for analysis (discovery stage: 2154 cases, 4308 controls).^29^ That study was subdivided into four subgroups based on the clinical syndrome (SD, agrammatic primary progressive aphasia, behavioural variant FTD and FTD with motor neuron disease). The diagnoses of FTD and its subtypes were based on clinical assessment (by specialized neurologists) and consensus clinical criteria.^41,42^ Of the included FTD cases, 70 patients had a pathologically confirmed frontotemporal lobar degeneration diagnosis. GWAS summary statistics for the whole FTD cohort and for SD (*n* = 308 cases, 616 controls) were obtained from: https://rdr.ucl.ac.uk/articles/dataset/IFGC_Summary-statistics_Data-sharing/13042166.

The largest LBD GWAS (*n* = 2981 cases, 4391 controls) was included as another outcome.^30^ That GWAS enrolled only European ancestry participants across 44 consortia. The LBD diagnosis was made based on the consensus criteria.^43,44^ Five independent genetic loci were associated with LBD at genome-wide significance. Summary-level statistics were obtained from the GWAS Catalog portal (https://www.ebi.ac.uk/gwas/).

### Positive control analyses

To test the validity of the genetic proxy of the exposures, we tested the association of the Global Connectivity of the Central_executive; Salience; Default_mode network (Net_Edge_ICA2) with major depressive disorder (MDD). The literature suggests that MDD is robustly associated with rsfMRI changes in this network^45^ and thus we tested if the causal association of this network rsfMRI connectivity and MDD risk.

For MDD outcome, we selected a recent meta-GWAS that included a total of 246 363 cases and 561 190 controls.^46^ Summary statistics were available from combined data set from Psychiatric Genomics Consortium (PGC) and UKB (*n* = 170 756 cases/329 443 controls) and not from 23andMe.^46^ The UKB MDD diagnosis was self-reported as ‘problems with nerves, anxiety, tension or depression’ (termed ‘broad depression’), while the PGC cohort MDD diagnoses were based on a range of depression phenotypes, including structured clinical interview as well as broader criteria. One hundred two independent SNPs were associated with MDD in the meta-analysis. MDD GWAS summary statistics were obtained from the PGC online database (https://www.med.unc.edu/pgc/download-results/).

### MR analyses

R-studio v1.3.1093 and the TwoSampleMR v0.5.5 package were used to perform the MR analyses. Genetic associations with exposures and outcomes were harmonized by aligning beta coefficients to the same effect allele. To assess the effect of the genetically proxied functional connectivity on the outcomes, the inverse-variance weighted (IVW) method was employed as the main MR analysis, due to being the more robust MR method.^22,47,48^ The adjusted *P* value for the level of significant results was assigned based on the number of tests performed (0.05/26 = 0.0019). Second, we performed MR model-based sensitivity analyses that are more robust to pleiotropy, including: MR-egger, weighted median, weighted mode (Supplementary Tables 5–8).^47,49^ For the significant associations, we investigated the genetic instruments SNPs for pleiotropy via assessing if those SNPs were implicated in the risk of the outcome, or in LD with SNPs implicated in the risk of the outcome. The LD analysis was completed using Ensembl LD Calculator including European ancestry data and selecting a stringent LD cut off (*R*^2^ > 0.001) for SNPs in the same chromosome.

### Power analyses

We calculated the power of the MR analyses based on the available GWAS cohorts to detect a significant association between the exposures and outcomes. First, we calculated the percentage of variance explained by the genetic proxy of the exposure based on this equation [2 × (beta exposure)^2^ × minor allele frequency × (1 − minor allele frequency)] (each SNP was individually calculated then results were added for all the SNPs).^50^ Then, using MR power calculator (https://shiny.cnsgenomics.com/mRnd/) we calculate the power of the MR analyses for a binary outcome prediction for three different OR cut offs (1.1, 1.25, 1.5).

## Results

A schematic illustration of the selected exposures and outcomes in the MR analyses is shown in Fig. 2. The MR IVW analyses did not show any significant association for the tested exposures with the outcomes after correcting for multiple testing (*P* > 0.0019; Table 3). Two resting state connectivity exposures in the visual cortex (implicating nodes in Calcarine and Lingual regions) showed a nominal level of association with LBD (*P* = 0.01), although not passing the multiple testing threshold. These nominally significant associations suggest that increased fluctuations amplitude in two occipital nodes could increase the risk for LBD (OR = 1.23, 1.18). Sensitivity analyses were in line with the primary analyses (Supplementary Tables 5–8).

**Table 3:** MR estimates for the effect of genetically proxied intrinsic functional networks features on risk of several dementias

Exposure category	Associated outcomes	Exposure Node/edge	N SNPs	Beta	Standard error	*P* value^a^
DMN, parieto-temporal	Ad	Net25_Node20	7	−0.01	0.07	0.91
		Net100_Node5	2	−0.08	0.25	0.76
		Net100_Node11	2	0.10	0.07	0.18
		Net100_Node24	3	0.00	0.11	0.98
		Net100_Node36	5	−0.12	0.08	0.15
		Net100_Node39	3	0.02	0.10	0.83
		Net100_Node49	3	−0.12	0.08	0.11
		Net25_Node9	9	−0.03	0.09	0.72
		Net100_Pair5_45	2	−0.04	0.09	0.65
		Net_Edge_ICA2	7	−0.07	0.06	0.24
Frontal	FTD	Net25_Node16	8	0.18	0.20	0.36
		Net100_Node7	5	0.41	0.30	0.18
		Net100_Node13	7	0.43	0.22	0.05
		Net100_Node15	6	−0.08	0.23	0.74
		Net100_Node21	4	0.17	0.43	0.68
		Net100_Node31	3	0.52	0.38	0.17
		Net100_Node37	7	0.10	0.24	0.67
		Net100_Node40	3	0.10	0.33	0.76
		Net100_Node45	2	−0.10	0.39	0.79
Temporal pole	SD	Net100_Node9	3	−1.93	1.11	0.08
		Net100_Node43	2	0.02	−1.42	0.99
Occipital	LBD	Net100_Node4	2	−0.36	0.53	0.50
		Net100_Node8	2	1.23	0.48	0.01
		Net100_Node10	3	0.05	0.38	0.89
		Net100_Node38	4	0.14	0.54	0.80
		Net100_Node1	2	1.18	0.46	0.01

Ad, Alzheimer’s dementia; DMN, default mode network; FTD, Frontotemporal dementia; LBD, Lewy body dementia; SD, Semantic Dementia; SNP, single nucleotide polymorphism.

^a^Adjusted *P* value level of significance = 0.0019.

We sought to explore the implicated genetic variants in this association between visual cortex connectivity and LBD. SNP-level MR analyses (Supplementary Table 9) showed the genetic variants (rs10735666, rs10747064) that drove this association for Net100_Node8 and Net100_Node1 with LBD. Those variants are in the vicinity of *INPP5A,* a gene implicated in calcium signaling and in the risk for Parkinson disease.^51^

As a secondary analysis, we ran IVW MR for the 3 SNPs associated with Visual 1 network connectivity and LBD. We found that increased within-network functional connectivity was associated with lower risk for LBD (Table 4). We then ran LD analysis between those 3 SNPs and the 5 SNPs associated with LBD to test for pleiotropy. LD analyses showed that none of the Visual 1 network connectivity SNPs are in LD with LBD SNPs.

**Table 4:** Visual 1 network rsfMRI connectivity and risk for LBD

Exposure category	Associated outcomes	Exposure Node/edge	N SNPs	Beta	Standard error	*P* value
Occipital	LBD	Visual 1 network	2	−1.1	0.52	0.03

LBD, Lewy body dementia; SNP, single nucleotide polymorphism.

To test the validity of the genetic instruments of the exposures, we tested their association with the outcome of MDD as a positive control. We selected the exposure Global Connectivity of the Central_executive; Salience; Default_mode network (Net_Edge_ICA2) and tested it for causal association with MDD via IVW MR approach. We found a significant association between connectivity in this network and the risk for MDD (Table 5). Supplementary Table 10 provides odds ratio and confidence intervals for associations between rsfMRI and LBD as well as rsfMRI and MDD.

**Table 5:** The association between DMN, parieto-temporal global connectivity and MDD

Exposure category	Associated outcomes	Exposure Node/edge	N SNPs	Beta	Standard error	*P* value
DMN, parieto-temporal	MDD	Net_Edge_ICA2	8	−0.07	0.03	0.024

DMN, default mode network; MDD, major depressive disorder; SNP, single nucleotide polymorphism.

The power analyses showed that the MR analyses were overall well-powered to detect a significant association with low effect size (Supplementary Tables 11–14). The power analyses showed high power to detect an association with Ad (larger sample size), and relatively moderate power for FTD and LBD. The power analyses showed low power to detect association with SD.

## Discussion

Leveraging human genomic data, we provide robust and novel data regarding the causal effects of intrinsic functional networks on neurodegeneration. Overall, our comprehensive MR analyses did not support a casual association between intrinsic functional networks connectivity patterns and neurodegenerative diseases. Instead, our results support the notion that any changes in rsfMRI patterns seen in patients with cognitive neurodegenerative diseases are secondary to the disease process, which can start decades before the symptoms onset^52,53^ and not a risk factor for it. In addition, our results suggest that even if the rsfMRI changes were seen before the onset of symptoms or the detection of disease biomarkers (e.g. amyloid changes in brain), those changes are likely due to non-detectable disease process changes, which may be missed due to the low sensitivity of our current technologies. Furthermore, it is also possible that the genetic variants of the neurodegenerative disease itself might be playing a role in affecting the rsfMRI patterns (e.g. as in the case of APOE4),^54,55^ in which early rsfMRI changes are due to shared genetic etiology with neurodegeneration. Although the lack of association overall between rsfMRI phenotypes and neurodegeneration outcomes could mean lack of causal link, other possible explanations should be considered. The Ad analysis had sufficient power; however, the other neurodegenerative outcomes had relatively reduced power to detect an effect, suggesting larger sample sizes may be needed to assess these relationships. Our findings are in line with current Ad literature, in which rsfMRI changes in autosomal dominant Ad carriers were nascent in preclinical stages (with positive Ad biomarkers) and became magnified in more advanced stages of disease.^56^ Furthermore, accumulating evidence has shown that amyloid-β and tau deposition are associated with decreased relevant hub connectivity, supporting the notion that the disease pathology causes functional connectivity changes.^57^ Thus, our results further support the hypothesis that the pathology of Ad damages structural and functional brain network connectivity, which correlates with cognitive impairment.^57,58^ Other neurodegenerative diseases are less studied, but similar findings are seen in FTD, in which disease progression was associated with decreased rsfMRI connectivity.^59,60^ In LBD, structural and functional connectivity changes in the occipital cortex have been correlated with visual hallucinations.^61^ In SD, patients with known temporal pole atrophy have decreased functional connectivity in the ventral semantic network involving anterior middle temporal and angular gyri, which correlates with weak semantic performance.^62^

The nominally significant associations with LBD are of interest, albeit not withstanding multiple testing corrections. The main analysis showed that increased fluctuation amplitude in two occipital nodes is associated with increased risk of LBD. However, the genetic variants driving this association are in a gene implicated in Parkinson disease risk, which suggests that pleiotropy is biasing this analysis. To further evaluate this association, we found that increased network functional connectivity within the visual cortex was associated with lower risk for LBD, which provided additional evidence for the potential association between visual networks functional connectivity and risk for LBD. These results need to be replicated and to be further studied in large longitudinal rsfMRI studies to assess if alterations in visual network connectivity can be a risk factor for LBD. One potential mechanism linking changes in visual network connectivity and occipital lobe neurodegeneration (as seen in LBD) is transneuronal degeneration, which has been suggested in prior neuroimaging work associating visual impairment and brain neurodegeneration.^63^ Transneuronal degeneration is a neurodegeneration process that evolves over time in which the dysfunction of one neuron causes the degeneration of its postsynaptic (anterograde) or presynaptic (retrograde) neuron.^64^

This study has multiple strengths. First, is the use of a large cohort (*n* = 47 276) for identifying the genetic proxy for the neuroimaging phenotypes, and the use of the largest GWAS cohorts to date for the neurodegenerative disease outcomes. This facilitated well-powered MR analyses especially for Ad. Second, is the use of a stringent cut off for the level of association between the genetic variants and exposures of interest (at the significance level 2.8 × 10^−11^). Third, is the use of MR approach allows for testing the causal effect of the exposures on the outcomes, and is less confounded by reverse causality. Fourth, is the consistency of the findings across multiple lines of sensitivity analyses. Fifth, is the ability to complete comprehensive analyses of many nodes in networks implicated in four types of neurodegenerative disorders. Sixth, is the use of a positive control analysis to assess the validity of the exposures further supports our results. We also acknowledge a number of limitations that require caution in interpreting the results of this study. The sample size of the SD GWAS cohort (keeping in mind it is a rare disease) was relatively small, which greatly limited power. In addition, these analyses were conducted using data from European populations, and therefore these findings may not generalize to other ancestry groups. Moreover, our results cannot rule out a causal effect between sporadic (i.e. non-genetically driven) changes in intrinsic functional connectivity and neurodegeneration disorders. Future MR studies should leverage upcoming GWAS studies of rsfMRI features that include larger or independent cohorts than the ones we used in this study in an attempt to further evaluate and replicate our results. Finally, rsfMRI studies have limitations in how much they can explain or detect connectivity alterations, and thus other technologies (such as EEG changes or their genetic proxies) to assess connectivity patterns as a potential risk for neurodegeneration should be leveraged in future studies.

In conclusion, our comprehensive MR analyses answered for the first time an important question regarding the possible causal role of resting state intrinsic functional networks alterations on neurodegeneration. Our MR results support the notion that resting state intrinsic functional network alterations are likely secondary to the neurodegeneration process and not causal for it. Nonetheless, the reported nominal association between the visual network connectivity and LBD requires further evaluation.

## Supplementary Material

fcaf098_Supplementary_Data

## Data Availability

This study generated data are all included in the manuscript or supplemental materials. The summary statistics of the GWAS studies are publicly available in their correlated publications.

## References

[1] Collaborators GBDN . Global, regional, and national burden of neurological disorders, 1990–2016: A systematic analysis for the Global Burden of Disease Study 2016. Lancet Neurol. 2019;18(5):459–480. 30879893 10.1016/S1474-4422(18)30499-X PMC6459001

[2] Diseases GBD, Injuries C. Global burden of 369 diseases and injuries in 204 countries and territories, 1990–2019: A systematic analysis for the Global Burden of Disease Study 2019. Lancet. 2020;396(10258):1204–1222. 33069326 10.1016/S0140-6736(20)30925-9 PMC7567026

[3] Perovnik M, Rus T, Schindlbeck KA, Eidelberg D. Functional brain networks in the evaluation of patients with neurodegenerative disorders. Nat Rev Neurol. 2023;19(2):73–90. 36539533 10.1038/s41582-022-00753-3

[4] Jones DT, Knopman DS, Gunter JL, et al Cascading network failure across the Alzheimer's disease spectrum. Brain. 2016;139(Pt 2):547–562. 26586695 10.1093/brain/awv338 PMC4805086

[5] Buckner RL, Sepulcre J, Talukdar T, et al Cortical hubs revealed by intrinsic functional connectivity: Mapping, assessment of stability, and relation to Alzheimer's disease. J Neurosci. 2009;29(6):1860–1873. 19211893 10.1523/JNEUROSCI.5062-08.2009 PMC2750039

[6] Buckner RL, DiNicola LM. The brain's default network: Updated anatomy, physiology and evolving insights. Nat Rev Neurosci. 2019;20(10):593–608. 31492945 10.1038/s41583-019-0212-7

[7] Malagurski B, Deschwanden PF, Jancke L, Merillat S. Longitudinal functional connectivity patterns of the default mode network in healthy older adults. Neuroimage. 2022;259:119414. 35760292 10.1016/j.neuroimage.2022.119414

[8] Staffaroni AM, Brown JA, Casaletto KB, et al The longitudinal trajectory of default mode network connectivity in healthy older adults varies as a function of age and is associated with changes in episodic memory and processing speed. J Neurosci. 2018;38(11):2809–2817. 29440553 10.1523/JNEUROSCI.3067-17.2018 PMC5852659

[9] Jones DT, Machulda MM, Vemuri P, et al Age-related changes in the default mode network are more advanced in Alzheimer disease. Neurology. 2011;77(16):1524–1531. 21975202 10.1212/WNL.0b013e318233b33d PMC3198977

[10] Fu H, Hardy J, Duff KE. Selective vulnerability in neurodegenerative diseases. Nat Neurosci. 2018;21(10):1350–1358. 30250262 10.1038/s41593-018-0221-2 PMC6360529

[11] Mattsson N, Schott JM, Hardy J, Turner MR, Zetterberg H. Selective vulnerability in neurodegeneration: Insights from clinical variants of Alzheimer's disease. J Neurol Neurosurg Psychiatry. 2016;87(9):1000–1004. 26746185 10.1136/jnnp-2015-311321

[12] Rogalski E, Weintraub S, Mesulam MM. Are there susceptibility factors for primary progressive aphasia? Brain Lang. 2013;127(2):135–138. 23489582 10.1016/j.bandl.2013.02.004 PMC3740011

[13] Miller ZA, Spina S, Pakvasa M, et al Cortical developmental abnormalities in logopenic variant primary progressive aphasia with dyslexia. Brain Commun. 2019;1(1):fcz027. 32699834 10.1093/braincomms/fcz027 PMC7364264

[14] Weintraub S, Rader B, Coventry C, et al Familial language network vulnerability in primary progressive aphasia. Neurology. 2020;95(7):e847–e855. 32699140 10.1212/WNL.0000000000009842 PMC7605508

[15] Heeger DJ, Ress D. What does fMRI tell us about neuronal activity? Nat Rev Neurosci. 2002;3(2):142–151. 11836522 10.1038/nrn730

[16] de Wilde MC, Overk CR, Sijben JW, Masliah E. Meta-analysis of synaptic pathology in Alzheimer's disease reveals selective molecular vesicular machinery vulnerability. Alzheimers Dement. 2016;12(6):633–644. 26776762 10.1016/j.jalz.2015.12.005 PMC5058345

[17] Overk CR, Masliah E. Pathogenesis of synaptic degeneration in Alzheimer's disease and Lewy body disease. Biochem Pharmacol. 2014;88(4):508–516. 24462903 10.1016/j.bcp.2014.01.015 PMC3973539

[18] Gao C, Jiang J, Tan Y, Chen S. Microglia in neurodegenerative diseases: Mechanism and potential therapeutic targets. Signal Transduct Target Ther. 2023;8(1):359. 37735487 10.1038/s41392-023-01588-0 PMC10514343

[19] Rajendran L, Paolicelli RC. Microglia-mediated synapse loss in Alzheimer's disease. J Neurosci. 2018;38(12):2911–2919. 29563239 10.1523/JNEUROSCI.1136-17.2017 PMC6596066

[20] Fox MD, Halko MA, Eldaief MC, Pascual-Leone A. Measuring and manipulating brain connectivity with resting state functional connectivity magnetic resonance imaging (fcMRI) and transcranial magnetic stimulation (TMS). Neuroimage. 2012;62(4):2232–2243. 22465297 10.1016/j.neuroimage.2012.03.035 PMC3518426

[21] Sheline YI, Morris JC, Snyder AZ, et al APOE4 allele disrupts resting state fMRI connectivity in the absence of amyloid plaques or decreased CSF Abeta42. J Neurosci. 2010;30(50):17035–17040. 21159973 10.1523/JNEUROSCI.3987-10.2010 PMC3023180

[22] Sanderson E, Glymour MM, Holmes MV, et al Mendelian randomization. Nat Rev Methods Primers. 2022;2(1):6pp. 10.1038/s43586-021-00092-5 PMC7614635 37325194

[23] Davies NM, Holmes MV, Smith GD. Reading Mendelian randomisation studies: A guide, glossary, and checklist for clinicians. BMJ. 2018;362:k601. 30002074 10.1136/bmj.k601 PMC6041728

[24] Gagnon E, Daghlas I, Zagkos L, et al Mendelian randomization applied to neurology: Promises and challenges. Neurology. 2024;102(4):e209128. 38261980 10.1212/WNL.0000000000209128 PMC7615637

[25] Anderson KM, Ge T, Kong R, et al Heritability of individualized cortical network topography. Proc Natl Acad Sci U S A. 2021;118(9):e2016271118. 33622790 10.1073/pnas.2016271118 PMC7936334

[26] J POFTG, Sprooten E, Beckmann CF, Franke B, Bralten J. Shared genetic influences on resting-state functional networks of the brain. Hum Brain Mapp. 2022;43(6):1787–1803. 35076988 10.1002/hbm.25712 PMC8933256

[27] Zhao B, Li T, Smith SM, et al Common variants contribute to intrinsic human brain functional networks. Nat Genet. 2022;54(4):508–517. 35393594 10.1038/s41588-022-01039-6 PMC11987081

[28] Bellenguez C, Kucukali F, Jansen IE, et al New insights into the genetic etiology of Alzheimer's disease and related dementias. Nat Genet. 2022;54(4):412–436. 35379992 10.1038/s41588-022-01024-z PMC9005347

[29] Ferrari R, Hernandez DG, Nalls MA, et al Frontotemporal dementia and its subtypes: A genome-wide association study. Lancet Neurol. 2014;13(7):686–699. 24943344 10.1016/S1474-4422(14)70065-1 PMC4112126

[30] Chia R, Sabir MS, Bandres-Ciga S, et al Genome sequencing analysis identifies new loci associated with Lewy body dementia and provides insights into its genetic architecture. Nat Genet. 2021;53(3):294–303. 33589841 10.1038/s41588-021-00785-3 PMC7946812

[31] Skrivankova VW, Richmond RC, Woolf BAR, et al Strengthening the reporting of observational studies in epidemiology using Mendelian randomisation (STROBE-MR): Explanation and elaboration. BMJ. 2021;375:n2233. 34702754 10.1136/bmj.n2233 PMC8546498

[32] Perovnik M, Tomse P, Jamsek J, et al Identification and validation of Alzheimer's disease-related metabolic brain pattern in biomarker confirmed Alzheimer's dementia patients. Sci Rep. 2022;12(1):11752. 35817836 10.1038/s41598-022-15667-9 PMC9273623

[33] Perovnik M, Tomse P, Jamsek J, Tang C, Eidelberg D, Trost M. Metabolic brain pattern in dementia with Lewy bodies: Relationship to Alzheimer's disease topography. Neuroimage Clin. 2022;35:103080. 35709556 10.1016/j.nicl.2022.103080 PMC9207351

[34] Ibach B, Poljansky S, Marienhagen J, Sommer M, Manner P, Hajak G. Contrasting metabolic impairment in frontotemporal degeneration and early onset Alzheimer's disease. Neuroimage. 2004;23(2):739–743. 15488423 10.1016/j.neuroimage.2004.06.041

[35] Harper L, Bouwman F, Burton EJ, et al Patterns of atrophy in pathologically confirmed dementias: A voxelwise analysis. J Neurol Neurosurg Psychiatry. 2017;88(11):908–916. 28473626 10.1136/jnnp-2016-314978 PMC5740544

[36] Hohenfeld C, Werner CJ, Reetz K. Resting-state connectivity in neurodegenerative disorders: Is there potential for an imaging biomarker? Neuroimage Clin. 2018;18:849–870. 29876270 10.1016/j.nicl.2018.03.013 PMC5988031

[37] Rolls ET, Huang CC, Lin CP, Feng J, Joliot M. Automated anatomical labelling atlas 3. Neuroimage. 2020;206:116189. 31521825 10.1016/j.neuroimage.2019.116189

[38] Yamazaki Y, Zhao N, Caulfield TR, Liu CC, Bu G. Apolipoprotein E and Alzheimer disease: Pathobiology and targeting strategies. Nat Rev Neurol. 2019;15(9):501–518. 31367008 10.1038/s41582-019-0228-7 PMC7055192

[39] Bell S, Tozer DJ, Markus HS. Genome-wide association study of the human brain functional connectome reveals strong vascular component underlying global network efficiency. Sci Rep. 2022;12(1):14938. 36056064 10.1038/s41598-022-19106-7 PMC9440133

[40] Finn ES, Shen X, Scheinost D, et al Functional connectome fingerprinting: Identifying individuals using patterns of brain connectivity. Nat Neurosci. 2015;18(11):1664–1671. 26457551 10.1038/nn.4135 PMC5008686

[41] Neary D, Snowden JS, Gustafson L, et al Frontotemporal lobar degeneration: A consensus on clinical diagnostic criteria. Neurology. 1998;51(6):1546–1554. 9855500 10.1212/wnl.51.6.1546

[42] Rascovsky K, Hodges JR, Knopman D, et al Sensitivity of revised diagnostic criteria for the behavioural variant of frontotemporal dementia. Brain. 2011;134(Pt 9):2456–2477. 21810890 10.1093/brain/awr179 PMC3170532

[43] McKeith IG, Boeve BF, Dickson DW, et al Diagnosis and management of dementia with Lewy bodies: Fourth consensus report of the DLB Consortium. Neurology. 2017;89(1):88–100. 28592453 10.1212/WNL.0000000000004058 PMC5496518

[44] Emre M, Aarsland D, Brown R, et al Clinical diagnostic criteria for dementia associated with Parkinson's disease. Mov Disord. 2007;22(12):1689–1707. 17542011 10.1002/mds.21507

[45] Yan CG, Chen X, Li L, et al Reduced default mode network functional connectivity in patients with recurrent major depressive disorder. Proc Natl Acad Sci U S A. 2019;116(18):9078–9083. 30979801 10.1073/pnas.1900390116 PMC6500168

[46] Howard DM, Adams MJ, Clarke TK, et al Genome-wide meta-analysis of depression identifies 102 independent variants and highlights the importance of the prefrontal brain regions. Nat Neurosci. 2019;22(3):343–352. 30718901 10.1038/s41593-018-0326-7 PMC6522363

[47] Hemani G, Zheng J, Elsworth B, et al The MR-Base platform supports systematic causal inference across the human phenome. Elife. 2018;7:e34408. 29846171 10.7554/eLife.34408 PMC5976434

[48] Lin Z, Deng Y, Pan W. Combining the strengths of inverse-variance weighting and egger regression in Mendelian randomization using a mixture of regressions model. PLoS Genet. 2021;17(11):e1009922. 34793444 10.1371/journal.pgen.1009922 PMC8639093

[49] Burgess S, Smith GD, Davies NM, et al Guidelines for performing Mendelian randomization investigations. Wellcome Open Res. 2019;4:186. 32760811 10.12688/wellcomeopenres.15555.1 PMC7384151

[50] Valdes-Marquez E, Parish S, Clarke R, et al Relative effects of LDL-C on ischemic stroke and coronary disease: A Mendelian randomization study. Neurology. 2019;92(11):e1176–e1187. 30787162 10.1212/WNL.0000000000007091 PMC6511103

[51] Cao M, Park D, Wu Y, De Camilli P. Absence of Sac2/INPP5F enhances the phenotype of a Parkinson's disease mutation of synaptojanin 1. Proc Natl Acad Sci U S A. 2020;117(22):12428–12434. 32424101 10.1073/pnas.2004335117 PMC7275725

[52] Wang L, Brier MR, Snyder AZ, et al Cerebrospinal fluid Abeta42, phosphorylated Tau181, and resting-state functional connectivity. JAMA Neurol. 2013;70(10):1242–1248. 23959173 10.1001/jamaneurol.2013.3253 PMC3836828

[53] Sheline YI, Raichle ME, Snyder AZ, et al Amyloid plaques disrupt resting state default mode network connectivity in cognitively normal elderly. Biol Psychiatry. 2010;67(6):584–587. 19833321 10.1016/j.biopsych.2009.08.024 PMC2829379

[54] Machulda MM, Jones DT, Vemuri P, et al Effect of APOE epsilon4 status on intrinsic network connectivity in cognitively normal elderly subjects. Arch Neurol. 2011;68(9):1131–1136. 21555604 10.1001/archneurol.2011.108 PMC3392960

[55] Dennis NA, Browndyke JN, Stokes J, et al Temporal lobe functional activity and connectivity in young adult APOE varepsilon4 carriers. Alzheimers Dement. 2010;6(4):303–311. 19744893 10.1016/j.jalz.2009.07.003 PMC2891943

[56] Chhatwal JP, Schultz AP, Johnson KA, et al Preferential degradation of cognitive networks differentiates Alzheimer's disease from ageing. Brain. 2018;141(5):1486–1500. 29522171 10.1093/brain/awy053 PMC5917745

[57] Yu M, Sporns O, Saykin AJ. The human connectome in Alzheimer disease—Relationship to biomarkers and genetics. Nat Rev Neurol. 2021;17(9):545–563. 34285392 10.1038/s41582-021-00529-1 PMC8403643

[58] Brier MR, Thomas JB, Snyder AZ, et al Loss of intranetwork and internetwork resting state functional connections with Alzheimer's disease progression. J Neurosci. 2012;32(26):8890–8899. 22745490 10.1523/JNEUROSCI.5698-11.2012 PMC3458508

[59] Hafkemeijer A, Moller C, Dopper EG, et al A longitudinal study on resting state functional connectivity in behavioral variant frontotemporal dementia and Alzheimer's disease. J Alzheimers Dis. 2017;55(2):521–537. 27662284 10.3233/JAD-150695

[60] Ferreira LK, Lindberg O, Santillo AF, Wahlund LO. Functional connectivity in behavioral variant frontotemporal dementia. Brain Behav. 2022;12(12):e2790. 36306386 10.1002/brb3.2790 PMC9759144

[61] Mehraram R, Peraza LR, Murphy NRE, et al Functional and structural brain network correlates of visual hallucinations in Lewy body dementia. Brain. 2022;145(6):2190–2205. 35262667 10.1093/brain/awac094 PMC9246710

[62] Battistella G, Henry M, Gesierich B, et al Differential intrinsic functional connectivity changes in semantic variant primary progressive aphasia. Neuroimage Clin. 2019;22:101797. 31146321 10.1016/j.nicl.2019.101797 PMC6465769

[63] Garzone D, Finger RP, Mauschitz MM, et al Visual impairment and retinal and brain neurodegeneration: A population-based study. Hum Brain Mapp. 2023;44(7):2701–2711. 36852616 10.1002/hbm.26237 PMC10089094

[64] Fornito A, Zalesky A, Breakspear M. The connectomics of brain disorders. Nat Rev Neurosci. 2015;16(3):159–172. 25697159 10.1038/nrn3901

