# Supplementary material for: The causal association between resting state intrinsic functional networks and neurodegeneration
Source: Brain Commun. 2025 Mar 4;7(2):fcaf098. doi: 10.1093/braincomms/fcaf098 (PMC11913654; doi:10.1093/braincomms/fcaf098)
Supplement: fcaf098_Supplementary_Data [file fcaf098_supplementary_data.zip › Supplementary_Notes.docx]

**Supplemental Notes**

**rsfMRI data acquisition and processing**

In the rsfMRI GWAS study, the rsfMRI cohorts included a discovery sample from the UK biobank British participants (UKB n= 34,691), and validation samples of four European-ancestry GWAS^1^: (UKBW, n= 5,056), ABCDE (n= 3,821), HCP European (n= 495) and PNC (n= 510). For the discovery UKB cohort, the mean age was 62.51 with standard deviation 7.47 years, and 47% of participants were males. The rsfMRI datasets were separately processed following the procedures in UKB imaging pipeline^2^. Briefly, rsfMRI data were acquired at 490 time points and a duration of 6 minutes, each with a 2.4×2.4×2.4 mm spatial resolution. The analyses pipeline included image cleaning, image registration, and representative time series generation. The time series was parcellated into 25 and 100 spatially-independent nodes using group-level Independent Component Analysis (ICA). After removing components thought to be artifactual (components explained by motion/ heart rate/ respiration etc.) 21 and 55 nodes remained.

In the rsfMRI GWAS study, for every participant, three sets of phenotypes were calculated based on these 76 nodes: 1) Fluctuation Amplitude (76 per participant):  defined as the temporal standard deviation within a given component. 2) Functional Connectivity (1,695 per participant): defined as the synchronized connectivity between two nodes using ICA with dual regression. 3) Global Connectivity (6 per participant): defined as the top six components selected from the adjacency matrix of all nodes (1,695 edges) by principal-component analysis (PCA) and ICA ^1^.

In total, there were 1,777 rsfMRI traits/ phenotypes generated (including 76 fluctuation amplitude features (21+55), 1,695 functional connectivity features $\left( \frac{\text{55}^{\text{2}}\text{-55}}{\text{2}}\text{+}\frac{\text{21}^{\text{2}}\text{-21}}{\text{2}} \right)$, and 6 global connectivity features. The anatomical locations for nodes were defined based on their proximity to the AAL (automated anatomical labeling) atlas ^3^.

**Summary of the rsfMRI GWAS analyses**

Briefly, the discovery stage of the intrinsic functional network’s connectivity GWAS was completed for 1,777 intrinsic brain activity traits and 9,026,427 common genetic variants in a UKB European sample (n = 34,691). Genome-wide association analysis was performed using fastGWA and adjusted for the effects of age (at imaging), age-squared, sex, age–sex interaction, age-squared–sex interaction, imaging site, head location, head motion, head size, long-term drifts and the top 40 genetic PCs. At the significance level 2.8 × 10^−11^ (5 × 10^−8^/1,777), the study identified 241 lead independent genetic variants (linkage disequilibrium (LD) r2 < 0.1). Then, the study characterized 603 significant locus–trait associations with 191 traits (75 amplitude, 111 pairwise functional connectivity and 5 global functional connectivity) in 45 genomic regions. Then, the study performed validation and meta-analyses, which supported their genetic associations. The study also tested for association in non-European ancestry cohorts. Polygenic risk score analyses were then completed and showed overall consistency of genetic effects in European-ancestry cohorts but suggested that that there may be population-specific influences on brain function in non-European-ancestry cohorts. SNP heritability was assessed by GCTA using all autosomal SNPs in the UKB British cohort. Of interest, the tested neuroimaging phenotypes showed overall significant heritability (heritability (h2) estimates overall varying reaching up to 60%, while mean heritability of the amplitude traits was 27.5%).

**References:**

1. Zhao B, Li T, Smith SM, et al. Common variants contribute to intrinsic human brain functional networks. *Nat Genet.* 2022;54(4):508-517.

2. Alfaro-Almagro F, Jenkinson M, Bangerter NK, et al. Image processing and Quality Control for the first 10,000 brain imaging datasets from UK Biobank. *Neuroimage.* 2018;166:400-424.

3. Rolls ET, Huang CC, Lin CP, Feng J, Joliot M. Automated anatomical labelling atlas 3. *Neuroimage.* 2020;206:116189.
